# Antidiabetic and antioxidant activities of ethanolic extract of *Semecarpus anacardium* (Linn.) bark

**DOI:** 10.1186/s12906-015-0662-z

**Published:** 2015-04-29

**Authors:** Md Ashraf Ali, Mir Imam Ibne Wahed, Naznin Ara Khatune, Bytul Mokaddesur Rahman, Ranjan Kumar Barman, Md Rafiqul Islam

**Affiliations:** Department of Pharmaceutical Engineering & Drug Delivery Science, Graduate School of Integrated Pharmaceutical & Nutritional Sciences, University of Shizuoka, Shizuoka, 422-8526 Japan; Department of Pharmacy, University of Rajshahi, Rajshahi, 6205 Bangladesh; Department of Pharmacy, Mawlana Bhashani Science and Technology University, Tangail, 1902 Bangladesh; Department of Pharmacy, Jessore University of Science and Technology, Jessore, Bangladesh

**Keywords:** *Semecarpus anacardium (SA)*, Blood glucose, Lipid profile, Liver enzyme, Antidiabetic activity, Antioxidant activity

## Abstract

**Background:**

Diabetes mellitus is a global health problem and constantly increasing day by day. The number of diabetic people in world is expected to rise to 366 million in 2030. The available drugs for diabetes, insulin or oral hypoglycemic agents have one or more side effects and search for new antidiabetic drugs with minimal or no side effects from medicinal plants is a challenging for us. The present study was undertaken to investigate the antidiabetic and antioxidant activity of *Semecarpus anacardium* (Linn.) (abbreviated as SF)*.*

**Methods:**

The antidiabetic activity was determined by using alloxan-induced diabetic rats. After 15 days of treatment, serum biochemical parameters such as TC, TG, LDL, HDL, SGOT and SGPT were estimated. The survival rate, body weight, organ weight, liver glycogen and blood parameters (RBC and Hb) were also measured. The antioxidant activity was measured by DPPH free radical scavenging assay. Phytochemical screening, total phenolic and total flavonoid content were determined by using standard methods.

**Results:**

The results showed that the survival rate was 100% in rats of Group SA 400. The effect of extract on blood glucose level in Groups SA 100, SA 200 and SA 400 were dose-dependent throughout the treatment period. No significant changes in organ weight to body weight ratio were observed, liver weights significantly improved in Groups SA 200 and SA 400. The bark extract exhibited significant (p < 0.05) anti-diabetic activity with lowering TC, TG, LDL level dose-dependently and protected liver which may be partially explained by attenuation of SGOT and SGPT levels and increases liver glycogen. The percentage of Hb and RBC counts were negatively correlated with the doses of extracts. In DPPH scavenging assay, IC_50_ values of SA extract and ascorbic acid were found 72.24 μg/ml and 17.81 μg/ml, respectively. Phytochemical screening showed the presence of steroids, triterpenoids, flavonoids, glycosides, saponins, and tannins that were contribute to biological activity.

**Conclusions:**

These results indicated that stem barks of *S. anacardium* possess strong anti-diabetic and antioxidant potentials and support traditional medicinal use for the treatment of diabetes mellitus and good source for natural antioxidants.

## Background

Diabetes mellitus (DM) is a metabolic syndrome with multiple etiology, is characterized by chronic hyperglycemia together with disturbances in carbohydrate, protein and fat metabolism results from a decrease in circulating concentration of insulin (insulin deficiency), a decrease in the response of peripheral tissues to insulin (insulin resistance) or both [1]. DM is the commonest clinical disorder affecting nearly 10% of the population all over the world [2]. At present, there are an estimated 246 million people with diabetes in the world, of whom about 80% reside in developing countries [3]. In Bangladesh, the situation is the most vulnerable and it has been estimated that the number of diabetes will rise from 3.2 million in 2000 to 11.7 million by the year 2030. In 2005, WHO reported that around 1.1 million people were died of diabetic complicacy, among which 80% from developing countries and it has also been suggested that the death rate will increase up to 50% [4]. Diabetes is a global disease with a huge adverse impact on the health and mortality, particularly from cardiovascular disorders [5]. To avoid the risk of serious complications from diabetes such as heart and blood vessels diseases, controlling not only blood glucose levels but also lipid levels are necessary [6].

Patients with diabetes have lipid disorders and an increased risk of coronary heart disease, peripheral vascular disease and cerebrovascular disease [7,8]. Hyperlipidemia is a characteristics feature of drug-induced (alloxan or streptozotocin) diabetes in rats and rabbits as well as in poorly controlled diabetes in humans. Coronary heart disease (CHD) is the cause of about 50% of all deaths in the United States. The incidence of CHD is correlated with elevated levels of LDL cholesterol (LDL-CH) and triglycerols (TG) and with low levels of HDL cholesterol (HDL-CH). Hyperlipidemias can also results from genetic defects in lipoprotein metabolism or by a combination of genetic and lifestyle factors [9]. Dislipidemia and hyperhomocysteinemia are important factors associated with the early onset of atherosclerosis. Multiple mechanisms contribute to arterial disease (e.g. atherogenesis) in patients with type 2 diabetes. A high burden of abdominal fat presents the liver with elevated levels of free fatty acids through the portal circulation. This excess of free fatty acids will drive the overproduction of TG-rich lipoprotein particles, including VLDL-CH, LDL-CH, A reciprocal decrease in HDL accompanies hypertriglyceridemia characteristic of the type 2 diabetic state [10].

In addition to hyperglycemia, systemic or local elevations in insulin may contribute to aberrant lipid metabolism and vascular wall function. Imperfect normalization of glucose metabolism by replacement insulin therapy may alter the concentrations and compositions of potentially atherogenic lipoproteins [11,12]. Changes in the ratio of apolipoproteins A-I to A-II in HDL have been observed [13] possibly interfering with the protective role of these lipoproteins in vascular disease [14]. At the vascular wall, insulin may contribute directly to increasing the levels of cellular cholesterol via its ability to increase cellular sterol synthesis, induce LDL receptors, and inhibit HDL-mediated cholesterol removal [15]. It remains to be established whether the hyperinsulinemia is most detrimental to vascular health in diabetes. The alternative possibility is that hyperglycemia is associated with atherosclerosis.

*Semecarpus anacardium* (L.) is commonly known as Beula (Bangla), Bhallataka (Sanskrit), or Marking nut tree (English), belongs to the family Anacardiaceae [16]. In traditional medicine, it is used in the treatment of gout, rheumatic pain and cancer [17]. Previous studies has documented to posses immunomodulatory and anti-inflammatory [18], anti-arthritic [19] as well as anticancer activity [20]. Phytochemical studies revealed the presence of phenolic compounds as carbolic acid derivatives, bhilawanols, sterols, glycosides, bhilawanols, anacardic acid, anacardoside and flavonoids [21-23]. The nut of this plant has been reported on hypoglycemic and antihyperglycemic [24], hypocholesterolemic [25], antitumor [26], antioxidant [27] and hepatoprotective activity [28]. It also possesses cytotoxic, fungistatic and anti-lipid peroxidative properties [29-31]. Therefore, we determined the effects of ethanolic extract of *S. anacardium* stem barks on glucose, GOT, GPT, total cholesterol (TC) triglyceride (TG), LDL and HDL in the blood and glycogen content in liver on alloxan induced-diabetic rats and antioxidant activity by DPPH scavenging assay.

## Methods

### Plant materials

Fresh stem bark of the plant *Semecarpus anacardium* (Linn.) was collected from Madhupur forests, Tangail, April in 2009 and the plant authenticity was confirmed from the Bangladesh National Herbarium, Dhaka.

### Preparation of plant extract

The collected stem barks were washed and sun dried under shadow for several days. The dried stem barks were powdered in an electrical grinder after overnight drying in an oven below 50°C. The powdered plant barks were extracted with 95% ethanol at room temperature. The bottle were kept at room temperature and allowed to stand for several 7–10 days with occasional shaking and stirring. The extracts thus obtained were filtered through cotton and then through filter paper (Whatman Fitter Paper No. 1). The filtrate was defatted with petroleum ether for several times. Then, the defatted liquor was allowed to evaporate using rotary evaporator at temperature 40-45°C. Finally, a highly concentrated ethanol extract were obtained and kept in desiccators to dry to give a solid mass (1.67%).

### Drugs and chemicals

The standard drug, Metformin hydrochloride was the generous gift samples from Chemico Laboratories Ltd. Alloxan monohydrate was purchased from Sisco Research Laboratories Pvt. Ltd., Mumbai, India. Blood samples analyzed for blood glucose content by using BioLand G-423 glucose test meter (BioLand, Germany). Serum TC and TG concentrations were analyzed by measuring absorbance by UV-spectrophotometer (Shimadzu UV-1200, Tokyo, Japan), using wet reagent diagnostic kits (Boehringer Mannheim, GmbH, Germany). Serum LDL and HDL Cholesterol measured by blood analyzer (5000, Spain) using wet reagent diagnostic kits (Centronic GmbH Germany & Crescent Diagnostic Kits). SGOT and SGPT level were measured by using Crescent Diagnostic Kits and Human Gesellschaft fur Biochemical mbH Germany according to manufacturer’s protocol. DPPH (2, 2-diphenyl, 1-picrylhydrazyl), TCA (trichloroacetic acid), ferric chloride, Gallic acid and Quercetin were obtained from Sigma Chemical Co. USA. Ascorbic acid was obtained from SD Fine Chem. Ltd., Biosar, India. Folin-ciocalteu reagent was purchased from Merck, Germany. All chemicals and solvents were of reagent grade.

### Experimental animals

Nine-week-old male Long Evans rats (150-180 g) purchased from ICDDRB, Dhaka, Bangladesh and were housed in animals cages under standard environmental conditions (22-25°C, humidity 60-70%, 12 h light: 12 h dark cycle). The rats were fed with standard pellet diet obtained from ICDDRB, Dhaka and water ad libitum. The animals used in this study were cared in accordance with the guidelines on animal experimentation of our institute. The experimental procedures involving animals were conducted in accordance with the guidelines of Institute of Biological Sciences, University of Rajshahi, Bangladesh. The study protocol was approved by Institutional Animal, Medical Ethics, Biosafety and Biosecurity Committee (IAMEBBC) at the Institute of Biological Sciences, University of Rajshahi, Bangladesh.

### Induction of diabetes

After fasting 16 h, diabetes was induced into rats by in intra-peritoneal injection (i. p.) of alloxan monohydrade (120 mg/kg), dissolved in saline (100 μl/rat, ip.). After 72 h, plasma glucose levels were measured by glucometer using a blood sample from tail-vein of rat. Rat with blood sugar level higher than 11.5-13.5 mmol/l are considered as moderate diabetic. Age-matched normal healthy rat were used as normal control.

### Experimental design

In the experiment, a total of 30 long Evans rats were divided into following six groups for the oral administration of extracts/drugs or vehicle.

I. Normal Control (Group NC, Vehicle 0.5% methyl cellulose, op., n = 5)
II. Diabetic Control (Group DC, Vehicle 0.5% MC, op., n = 5)
III. Diabetic Standard, (Group DS, Metformin HCl, 150 mg/kg, op. n = 5)
IV. Diabetic + Extract 100 mg/kg (Group SA 100, op., n = 5)
V. Diabetic + Extract 200 mg/kg (Group SA 200, op., n = 5)
VI. Diabetic + Extract 400 mg/kg (Group SA 400, op., n = 5)

### Antidiabetic studies of SA extract on alloxan-induced diabetic rats

The animals of Group IV, Group V and Group VI received bark extract of *S. anacardium* at doses of 100, 200 and 400 mg/kg body weight once daily, for 15 days using intrgastric tube. Group III received metformin (150 mg/kg body weight), while Group II serves as diabetic control (received vehicle 0.5% MC). The blood samples were analyzed for blood glucose content by Glucometer at 0th, 5th 10th and 15th days of treatment.

### Measuring body weight and organ weight (liver, pancreases, kidney, heart, and lung)

The body weights of rats of each group were measured on 0, 3, 6, 9, 12 and 15 days during the treatment period. At the end of experiments, all the rats were anesthetized with diethyl ether, chest were opened and blood samples were withdrawn directly by heparinised syringes from aorta of heart and stored in test tube containing anticoagulant (EDTA). Then liver, kidney, pancreases, heart, and lung were removed and cleaned of the surrounding tissues. The organ weights (OW) were measured immediately and the ratio of organ weights to body weight ratio (O/B) were calculated and parts of them were stored in 10% formalin and in a refrigerator at −20°C for histopathology and biochemical estimations respectively.

### Collection of blood and serum and determination of serum total cholesterol (TC), triglycerides (TG), LDL-Ch, HDL-Ch, SGOT and SGPT

The blood samples were centrifuged at 4000 rpm for 20 minutes to separate serum by using an Ultra-Centrifuge Machine (Centurion, UK) and the serum was preserved at −20°C to examine Total Cholesterol (TC), Triglyceride (TG), LDL-cholesterol (LDL), HDL-cholesterol (HDL), SGOT and SGPT concentrations by UV Spectrophotometric method (Shimadzu UV-1200, Tokyo, Japan) using wet reagent diagnostic kits according to manufacturer’s protocol (Centronic GmbH Germany & Crescent Diagnostic Kits).

### Estimation of glycogen content in liver

The liver glycogen content was determined according to the method described by Tarnoky K. *et. al* [32]. Briefly, small portion of liver of all mice were extracted by treated with o-toluidine-glucose and after extraction; coupling reaction was done with trichloroacetic acid (TCA) and precipitation by alcohol and hydrolysis. Finally, estimation of glycogen was performed by UV spectrophotometer.

### *In vitro* antioxidant activity of SA extract by DPPH free radical scavenging assay

The scavenging effects of samples for DPPH (2, 2-diphenyl-1-picrylhydrazyl) free radical were monitored according to the method of Yen [33]. Accurately weight 0.004gm of DPPH and place it into a 100 ml of a volumetric flask. Then the volume is adjusted by methanol. Then the concentration of the solution is 0.004% of DPPH. The absorbance of this solution was taken at 517 nm against methanol as a blank and recorded as a control solution standard. Accurately weight 0.025gm of ascorbic acid and dissolved it into 5 ml of distilled water. The concentration of the solution is 5 μg/μl of ascorbic acid. This solution is called stock solution. Take 0.025gm of plant extract and dissolved it into 5 ml of methanol. The concentration of the solution is 5 μg/μl of plant extract. This solution is called stock solution. 200 μl of plant extract or standard of different concentration solution was taken in a test tube. 2 ml of reagent solution was added in test tube. Incubate the test tube for 30 mins to complete the reaction. Then the absorbance of the solution was measured at 517 nm against methanol as a blank by using UV spectrophotometer. A typical blank solution contained methanol. The mixture was mixed well and then left to stand in the dark for 30 min at room temperature, and its absorbance was read at 517 nm with a spectrophotometer against a blank. Trolox in the same concentrations was used as the positive control. All measurements were done in triplicate.

The percentage (%) of scavenging of the DPPH free radical was measured by using the following equation:

$$ \left\{\left({\mathrm{A}}_0\hbox{-} {\mathrm{A}}_1\right)/\ {\mathrm{A}}_0\right\}\times 100 $$

Where, A_0_ = absorbance of the control

A_1_ = absorbance of the extract/ standard

Then the percentage (%) of inhibition was plotted against log concentration and from the graph IC_50_ was calculated.

### Phytochemical screening tests

The phytochemical tests have been performed by the standard methods (Pollock and Stevens, 1965; Trease and Evans, 1996 and Plummer, 1985) [34-36].

### Determination of total phenolic compound content

Total phenol content in extract was determined by Folin-Ciocalteu reagent [37]. Extract (200 μg/ml) was mixed with 400 μl of the Folin-Ciocalteu reagent and 1.5 ml of20% sodium carbonate. The mixture was shaken thoroughly and made up to 10 ml with distilled water. The mixture was allowed to stand for 2 hrs. Then the absorbance at 765 nm was determined. The concentration of total phenol content in SA extract was then determined as mg of gallic acid equivalent by using an equation that was obtained from standard gallic acid graph.

### Determination of total flavonoids content

The total flavonoid content was determined using a method previously described by Kumaran K [38]. 1 ml of plant extract in ethanol (200 μg/ml) was mixed with 1 ml aluminium trichloride in ethanol (20 mg/ml) and a drop of acetic acid, and then diluted with ethanol to 25 ml. The absorption at 415 nm was read after 40 min. Blank samples were prepared from 1 ml of plant extract and a drop of acetic acid, and then diluted to 25 ml with ethanol. The total flavonoid content was determined using a standard curve of quercetin and expressed as mg of quercetin equivalent (QE/gm of extract dry materials).

### Statistical analysis

Data were expressed as mean ± Standard Error of Mean (SEM). Statistical comparison were performed by one-way (ANOVA) followed by Dunnett’s Multiple Comparison Test and the values were considered as statistically significant when p values were less than 0.05 (p < 0.05). Statistical calculations and the graph were prepared using GraphPad Prism Software version 5.0 (GraphPad Software, San Diego, CA, USA).

## Results

### Survival rate

The survival rate among the groups of rats with SA extract were 40%, 60% and 80% of 5 rats each in Group DC, Group SA 100 and Group SA 200 respectively, after 15 days treatment period. None of the rats died in Group DS, Group SA 400 and Group NC. The 15 days survival rate was significantly higher in Group SA 400 compared to Group DC (Table 1).

**Table 1:** **Survival rate of animals after 15 days of treatment with SA extract**

**Treatment and dose**	**Total Animals**	**Survivors**	**Deaths**	**Survival rat (%)**
Normal control (NC)	5	5	0	100
Diabetic Control (DC)	5	2	3	40
Diabetic standard (DS)	5	5	0	100 **
SA 100 (100 mg/kg)	5	3	2	60 *
SA 200 (200 mg/kg)	5	4	1	80 *
SA 400 (400 mg/kg)	5	5	0	100 **

Data expressed in percentages (%). (n = 5). (p < 0.001) Control group received 0.5% methyl cellulose and standard group received 150 mg/kg Metformin.

*p < 0.05, **p < 0.01 compared with diabetic control.

### Effect of SA extract on blood glucose levels in alloxan induced diabetic rats

**Figure 1:**
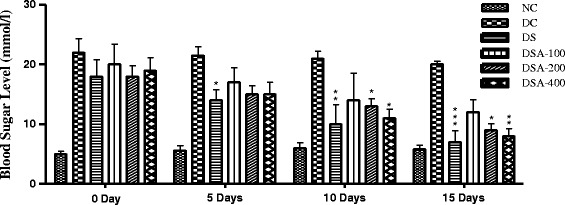
Time course of changes in blood sugar levels in the treatment period of 15 days. Data expressed in Mean ± Standard Error. (n = 5). Control group received 0.5% MC and standard group received 150 mg/kg Metformin, *p < 0.05 compared with diabetic control.

The blood glucose levels were significantly higher in Group DC compared to Group NC rats. The blood sugar levels in rats of Group SA100, Group SA 200 and Group SA 400 were lowered after 5, 10, 15 days of treatment. Group SA 200 and Group SA 400 rats showed significant glucose lowering efficacy between days 10–15 and were comparable to Group DS. However, Group SA 100 had no significant effect on blood glucose levels when to Group DS. After 15 days of treatment with extract glucose levels were significantly lowered in Group SA 100, SA 200 and SA 400 rats. The effect was dose-dependent and the most significant effect observed in Group SA 200 and Group SA 400 (p < 0.05) (Figure 1).

### Effect of SA extract on body weight changes and organ weight to body weight ratio

During the course of treatment, the significant changes in the body weight were not observed among the groups of rats which were shown in Table 2.

**Table 2:** **Effect of SA extract on body weight changes in normal and diabetic rats**

**Groups**	**0 Day**	**3 Days**	**6 Days**	**9 Days**	**12 Days**	**15 Days**
NC	174.2 ± 6.5	176.5 ± 4.23	178.4 ± 2.25	180.2 ± 4.32	183.8 ± 3.56	186.4 ± 5.78
DC	166.4 ± 3.9	167.4 ± 4.0	168.6 ± 4.2	170.4 ± 3.7	174.8 ± 3.8	175.0 ± 4.4
DS	156.6 ± 4.5	157.6 ± 4.2	157.8 ± 4.4	159.4 ± 5.7	163.1 ± 4.5	157.4 ± 3.2
SA 100	156.6 ± 4.4	152.8 ± 5.3	150.8 ± 7.5	150.4 ± 8.3	147.8 ± 9.4	146.2 ± 9.5
SA 200	153.4 ± 5.6	153.75 ± 4.6	154.51 ± 8.5	156.7 ± 3.3	163.5 ± 5.6	166.6 ± 6.0
SA 400	157.2 ± 6.5	158.2 ± 7.5	159.5 ± 5.3	161.5 ± 6.8	170.6 ± 3.4	172.4 ± 3.7

Data expressed in Mean ± Standard Error. (n = 5). Control group received 0.5% methyl cellulose and standard group received 150 mg/kg Metformin.

The results revealed that the heart weight, kidney weight, lung weight and pancreas weight did not significant changes after 15 days of treatment. Although the liver weight significantly decreased in Group DC, after treatment the values were normalized in Groups SA 200 and SA 400. No significant changes in organ weight to body weight ratio were observed among the experimental groups (Table 3).

**Table 3:** **Effect of SA extract on organ weight in alloxan diabetic rats (gm)**

**Group**	**Heart weight**	**Liver weight**	**Kidney Weight**	**Lung weight**	**Pancreas wt.**
NC	0.62 ± 0.05	4.87 ± 0.25	1.1 ± 0.06	0.38 ± 0.02	1.15 ± 0.07
DC	0.51 ± 0.04	3.35 ± 0.4	1.08 ± 0.0	0.39 ± 0.1	1.15 ± 0.5
DS	0.49 ± 0.03	4.32 ± 0.23*	0.98 ± 0	0.47 ± 0.04	1.35 ± 0.13
SA 100	0.54 ± 0.08	4.13 ± 0.33	0.97 ± 0.05	0.41 ± 0.41	1.27 ± 0.12
SA 200	0.52 ± 0.03	5.25 ± 0.3*	1.05 ± 0.06	0.52 ± 0.04	1.17 ± 0.2
SA 400	0.55 ± 0.16	5.47 ± 0.08*	1.05 ± 0.02	0.53 ± 0.07	1.29 ± 0.22

Data expressed in Mean ± Standard Error. (n = 5). *p < 0.05 compared with diabetic control.

### Effects of SA extract on lipid profile in alloxan diabetic rats

The effects of different doses of extract on lipid profile were shown in Table 4. The total cholesterol, triglycerides, LDL-CH significantly higher and HDL-CH were lowered in Group DC rats. Total cholesterol, triglycerides and LDL-CH levels in Group SA 100, Group SA 200 and Group SA 400 significantly decrease as compared to Group DC. However, HDL-CH was increased in treatment groups and all the effects were dose-dependent. Among the treatment groups Group SA 400 significantly improved dyslipidemia.

**Table 4:** **Effect of SA extract on lipid profile in normal and diabetic rats (mg/dl)**

**Group**	**Total Cholesterol (TC)**	**Triglycerides (TG)**	**LDL**	**HDL**
NC	168.5 ± 4.8	140 ± 2.1	110 ± 3.7	43 ± 1.4
DC	210 ± 3.4††	182 ± 3.9††	132 ± 4.2†	25 ± 3.0†
DS	155.5 ± 4.2**	135 ± 5.3**	105 ± 3.2*	40 ± 2.8*
SA 100	182 ± 4.6*	175 ± 2.6	125 ± 3.1	28 ± 1.7
SA 200	178 ± 3.8*	165 ± 4.3	121 ± 5.3	31 ± 2.1
SA 400	170 ± 5.6**	150 ± 2.8**	115 ± 2.5*	37 ± 1.9*

Data expressed in Mean ± Standard Error. (n = 5). *p < 0.05, and **p < 0.01 compared with diabetic control, †p < 0.05 and †† p < 0.01 compared with normal control.

### Effect of SA extract on liver glycogen content in normal and diabetic rats

**Figure 2:**
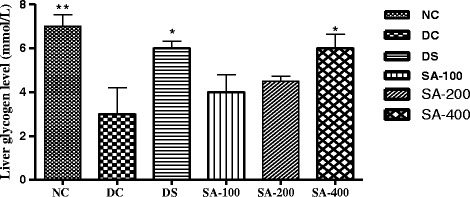
Effect of SA extract on liver glycogen content in normal and diabetic rats. Data expressed in Mean ± Standard Error. (n = 5). *p < 0.05 compared with diabetic control.

In this study it was found that the level of glycogen in liver is reduced in DS, DSA-100, DSA-200 and DSA-400 compared to normal control (NC) group. Treatment of diabetic rats with metformin and experimental groups significantly (p < 0.05) improved the level of glycogen content compared to DC group as shown in Figure 2.

### Effect of SA extract on SGOT and SGPT levels in alloxan diabetic rats

The effects of different doses of extract on SGOT and SGPT levels were shown in Table 5. Both the levels were significantly higher in Group DC rats. Oral ingestion of extracts reduced both the SGOT and SGPT levels in all treatment Group and was comparable to Group DC and protected liver which may be partially explained by the attenuation of SGOT and SGPT levels.

**Table 5:** **Effect of SA extract on SGOT and SGPT levels in alloxan diabetic rats**

**Group**	**SGOT (Unit/ml)**	**SGPT (Unit/ml)**
Normal Control (NC)	18 ± 4.02	21.5 ± 0.39
Diabetic Control (DC)	38 ± 2.83†	42.6 ± 0.6†
Standard (DS)	20.5 ± 3.07*	22.2 ± 0.51*
SA 100 (100 mg/kg)	27.0 ± 2.14*	29.3 ± 0.14*
SA 200 (200 mg/kg)	24.7 ± 3.5*	25.8 ± 0.32*
SA 400 (400 mg/kg)	22.4 ± 2.65*	23.1 ± 0.26*

Data expressed in Mean ± Standard Error. (n = 5). *p < 0.05 compared with diabetic control, †p < 0.05 compared with normal control.

### Effects of SA extract on blood parameters in alloxan diabetic rats

Although no significant differences were observed between Groups NC, DC and DS, treatment with extracts reduced Hb % and RBC counts in rats. The effects were negatively correlated with the doses of extract (Table 6).

**Table 6:** **Effects of** ***Semecarpus anacardium*** **bark extract on Hb (%) and RBC**

**Group**	**Hb % (mg/dl)**	**RBC (millions/mm** ^**3**^ **)**
Normal Control (NC)	13.3 ± 0.8	5.2 ± 0.1
Diabetic Control (DC)	12.6 ± 0.5	5.2 ± 0.1
Standard (DS)	12.5 ± 0.5	5.4 ± 0.04
SA 100 (100 mg/kg)	11.5 ± 0.5	4.8 ± 0.1
SA 200 (200 mg/kg)	10.4 ± 0.3†	4.8 ± 0.1†
SA 400 (400 mg/kg)	9.6 ± 0.6†*	4.7 ± 0.1*

Data expressed in Mean ± Standard Error. (n = 5). *p < 0.05 compared with diabetic control, †p < 0.05 compared with normal control.

### *In Vitro* antioxidant activity DPPH radical scavenging activity

**Figure 3:**
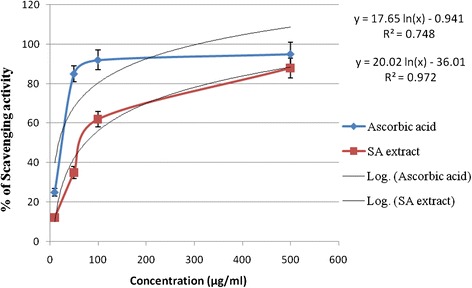
DPPH free radical scavenging activity (%) at various concentrations (μg/mL) of SA barks extract and ascorbic acid.

The free radical scavenging activity of SA extract has been evaluated by using the DPPH free radical. The antioxidant quality of an extract is determined by the IC_50_ value. The result of the DPPH scavenging activity of SA extract is shown in Figure 3. The extract exhibited DPPH radical scavenging activity with IC_50_ values of 72.24 μg/ml compared to ascorbic acid with IC_50_ 17.81 μg/ml.

### Phytochemical screening

Phytochemical analyses of the crude extract of *Semecarpus anacardium* revealed the presence of flavonoid, steroid, glycoside, saponin, tannins, and triterpinoid (Table 7).

**Table 7:** **Phytochemical test results of** ***S. anacardium*** **stem bark extract**

**Extract**	**Steroid**	**Alkaloid**	**Glycoside**	**Tannin**	**Triterpene**	**Saponin**	**Flavonoid**
EE of *S. anacardium*	+	-	+	+	+	+	+

EE: Ethanol-Extract; Sign (+) indicates present and sign (−) indicates absent.

### Determination of total phenolic compounds

**Figure 4:**
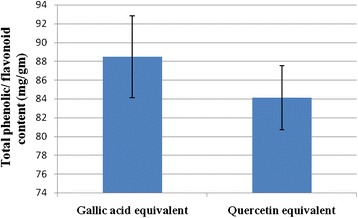
Total phenol and flavonoid content of SA extract represented as equivalent of gallic acid and quercetin respectively. Each values represented as means ± SE (n = 3).

The Folin-Ciocalteu reagent was used to estimate total phenols present in the extract and the value was expressed as Gallic Acid Equivalents (GAE). As shown in Figure 4, it was found that total phenolic content of the sample and calculated on the basis of the standard curve for gallic acid which was 88.47 ± 4.35 μgm gallic acid equivalents per gm of SA extract.

### Determination of total flavonoid content

The amount of total flavonoids determined by spectrophotometer is shown in Figure 4. The total flavonoid content was 84.13 ± 3.39 μgm of quercetin equivalent per gm of SA extract.

## Discussion

Diabetes is becoming the third “killer” of mankind after cancer and cardiovascular diseases, because of its high prevalence, morbidity and mortality [39]. The chronic hyperglycemia of diabetes is associated with long term damage, dysfunction and failure of various organs [40]. Hyperglycemia is an important factor in the development and progression of long-term complications of DM affecting kidney, retina, heart and nervous system. Patients with diabetes have lipid disorders (hypercholesterolemia and hypertriglyceridemia) and an increased risk of coronary heart disease, peripheral vascular disease and cerebrovascular disease. The hypoglycemic activity of many plants has been confirmed in hundreds of studies in experimental animals and several studies in diabetic patients. In Bangladesh, although a large number of medicinal plants have been tested for their anti-diabetic and anti-hyperlipidemic activity, many remain to be scientifically established.

The present investigation revealed that none of the rats died in Group DS, Group SA 400 and Group NC. The survival rate was significantly higher in Group SA 400 than that in Group DC (p < 0.05). The nuts showed no mortality when given in combinations with *Emblica officinalis* and honey as reported by Rajendran et. al [41]. The blood sugar levels in Group SA100, Group SA 200 and Group SA 400 were significantly (p < 0.05) reduced after 5, 10, 15 days of treatment. Group SA 200 and Group SA 400 rats showed significant glucose lowering efficacy between days 10–15 and the effects were dose-dependent and comparable to the effect of diabetic standard metformin. Our previous report for OGTT investigation of same extract showed that the ethanolic extract of *S. anacardium* reduced blood sugar significantly in glucose loaded hyperglycemic rats and produced more intense hypoglycemia in alloxan induced diabetic rats [42].

No significant changes in the body weight and organ weight to body weight ratio were observed among the treatment groups. Although the weight of liver significantly decreased, the weight of heart, kidney, lung and pancreas did not changed after 15 days of treatment. Treatment with extract improved liver weight significantly in Group SA 200 and SA 400. Oral Administration of extract significantly (p < 0.05) reduced the total cholesterol, triglycerides and LDL-CH levels and increased HDL-CH in Groups SA 100, SA 200 and SA 400 and all the effects were dose-dependent. Among the treatment groups Group SA400 significantly improved dyslipidemia. The ethanol extract of the plant *S. anacardium* bark exhibited significant antihyperglycemic and antihyperlipidemic activity in alloxan-induced diabetic rats, and the activity was comparable to that of the standard antidiabetic drug, Metformin HCl (150 mg/kg b. wt, op.).

A significant elevation of glycogen content was observed in all the treatment groups. However, the effect was most prominent in Group SA 400 rats. Similar observations were obtained by Sharma *et. al* with nuts of the same plant in cholesterol fed rabbits [25]. On the other hand, extract was reduced both the SGOT and SGPT levels in all treatment Group. Our observation showed that the extract at 100, 200 and 400 mg doses reduced Hb % and RBC. Similarly, it has been reported that nuts of the plant produced anemia at dose ranges 83.33-750 mg/kg [43]. However, no morphological disturbances of vital organs were observed at a dose of 500 mg/kg as reported by Rajendran et. al [41]. The stem barks extract protected liver which may be partially explained by the attenuation of SGOT and SGPT levels and increase in liver glycogen content.

The effect of antioxidants on DPPH radicals is thought to be due to their hydrogen donating ability [44]. Radical scavenging activities are very important to prevent the deleterious role of free radical in different diseases including diabetes. DPPH free radical scavenging is an accepted mechanism by which antioxidants act to inhibit lipid peroxidation. This method has been used extensively to predict antioxidant activities because of the relatively short time required for analysis. The DPPH radical scavenging activity of extract increased with increase in concentration (Figure 3). In DPPH assay, the extract showed a notable radical scavenging activity in a dose-dependent manner within a certain range and was significantly different (p < 0.05). The phytochemical screening of the plant *S. anacardium* stem bark showed the presence of steroids, triterpenoids, flavonoids, glycosides, saponins and tannins. Antioxidant activity is correlated with the total phenolic content and total flavonoids content in the extract that were likely to contribute to the radical scavenging activity. It is known that only flavonoids of a certain structure and particularly hydroxyl position in the molecule determine antioxidant properties, in general these properties depend on the ability to donate hydrogen or electron to a free radical.

The present investigation established the pharmacological evidence to support the folklore claim and the plant barks have antidiabetic and antioxidant activity. However, further investigations were warranted to isolate bioactive compounds from stem bark, to observe their antidiabetic and antioxidant effects and to find out the possible mechanism action for their beneficial effects both.

## Conclusion

The present study indicated that the stem barks of the plant *S. anacardium* possessed highest phenolic and flavonoid compound and exhibuted strong antidiabetic and antioxidant activities, which were comparable to the commercial antidibetic drug metformine and antioxidant ascorbic acid. This seems that the *Semecarpus anacardium* extract can be used as natural antidiabetic and antioxidant agent.

## Abbreviations

DM: Diabetes mellitus
SA: *Semecarpus anacardium*
ICDDRB: International Centre for Diarrhoeal Disease Research, Bangladesh
SGOT: Serum glutamate oxaloacetate transaminases
SGPT: Serum glutamate pyruvate transaminases
DPPH: 2, 2-diphenyl-1-picrylhydrazyl
TC: Total cholesterol
TG: Triglycerides
LDL: Low density lipoprotein
HDL: High density lipoprotein
